# Recyclable Aggregates of Mesoporous Titania Synthesized by Thermal Treatment of Amorphous or Peptized Precursors

**DOI:** 10.3390/ma11030381

**Published:** 2018-03-05

**Authors:** Maria Cristina Mascolo, Terry Arthur Ring

**Affiliations:** 1 Laboratory of Materials, Department of Civil and Mechanical Engineering, University of Cassino and Southern Latium, G. Di Biasio 43 Street, 03043 Cassino (FR), Italy; 2 Department of Chemical Engineering, University of Utah, Salt Lake City, UT 84112, USA; ring@eng.utah.edu

**Keywords:** amorphous and peptized precursors, peptizing HNO_3_, thermal crystallization, drying effect, micrometer aggregates, mesoporous titania

## Abstract

Recyclable aggregates of mesoporous titania with different anatase–rutile ratios have been prepared by thermal treatments of either amorphous or peptized precursors. These last two have been obtained by hydrolysis of either Ti(OC_2_H_5_)_4_ or of Ti(OC_2_H_5_)_4_ in mixture with 5 mol % Zr(OC_3_H_7_)_4_ at room temperature in the presence of NH_4_OH as a catalyzing agent. The anatase–rutile ratio, the recyclable aggregates of the nano-sized particles, the mesoporosity, the surface area and the crystallinity of the resulting crystallized products of titania can be controlled by the synthesis parameters including: concentration of ammonia catalyst, stirring time and concentration of the peptizing HNO_3_, drying method of peptized precursors, calcination temperature, and finally the ramp rate up to the titania crystallization temperature. A broad range of synthesis parameters control the crystal sizes of titania particles produced. This allows catalyst preparation with very different crystal size, surface area, anatase to rutile crystal ratio and various mesoporous structures. Drying by lyophilization of precursors reduce the aggregation of the primary particles giving micro-/macroporous structures.

## 1. Introduction 

Titania (TiO_2_) has attracted noticeable attention for numerous applications in various research areas including pigments, photo-catalysis, dye-sensitized solar cells, lithium ion batteries, sensor devices, protective coatings, sunscreen cosmetics and solar water splitting [1,2,3,4,5,6,7,8,9]. The effectiveness of TiO_2_ in practical applications relies upon its physical-chemical properties that depend on the synthesis of nano-sized titania particles. The efficiency of titania strongly depends on its structure in terms of crystallinity, morphology, crystallite size, porosity, surface area, doping and the presence of one or more polymorph phases [10,11,12,13,14,15].

The application of TiO_2_ powders in heterogeneous photo-catalysis reveals some drawbacks: the trouble to separate the powder from the aqueous dispersion making the recycling difficult and the tendency of the nano-particles to agglomerate leading to a reduction of the photo-catalytic reaction surface area [16]. On the other hand the immobilization of titania as a thin film reduces agglomeration but decreases the catalyst surface area [17,18,19]. In previous investigations mesoporous anatase samples, in the form of recyclable aggregates, have been prepared by hydrothermal treatments [20,21,22].

This work describes the synthesis of mesoporous titania aggregates by thermal treatments of either amorphous or crystalline precursors prepared at room temperature either by hydrolysis of either Ti(OC_2_H_5_)_4_ or Ti(OC_2_H_5_)_4_ in mixture with Zr(OC_3_H_7_)_4_ (5 mol %) and both catalysed with ammonia. The resulting xerogels and those peptized with HNO_3_ were submitted to thermal treatment that resulted in crystallization/coarsening. The experimental objective is to obtain recyclable aggregates of mesoporous titania with high values of both crystallinity and surface area, either as anatase form or as binary mixtures of the three different crystalline polymorphs of titania: anatase, rutile and brookite, respectively. It is well known that the co-presence of various crystallographic forms of TiO_2_ favours its photo-activity [23]. The Degussa commercial product P25 consists of a mixture of anatase and rutile, for example.

## 2. Experimental

The hydrolysis of Ti(OC_2_H_5_)_4_ (R.G., Sigma-Aldrich, Saint Louis, MO, USA) and mixtures of Ti(OC_2_H_5_)_4_ with Zr(OC_3_H_7_)_4_ (5 mol %) was performed at room temperature in ethanol obtaining the amorphous precursors of titania. According to the previous procedures [20], the hydrolysis treatments were carried out under continuous stirring and in the presence of different concentrations of NH_4_OH as catalysing agent, with reaction time of 1 h. The precipitates (0.05 mol) were washed three times with de-ionized water and separated by centrifugation. Some of these products were dried in an oven at 60 °C obtaining the corresponding xerogels. Other products were directly peptized under continuous stirring at 50 °C in the presence of 50 mL of aqueous solution of HNO_3_ 0.02 M or 0.1 M, using treatment times of 1 h or 3 h, respectively. It should be noted that adopting a peptizing temperature higher than 50 °C or a treatment time with 0.1 M HNO_3_ for longer than 3 h, or the presence of a more concentrated HNO_3_ solution results in the complete dissolution of the xerogel.

The peptized products, without washing with deionized water, were dried by lyophilization, using a Lio Cinquepascal apparatus (Milan, Italy), or at room temperature in the presence of silica gel or in an oven at 60 °C. Both peptized and un-peptized samples were thermally treated from 250 °C up to 600 °C for 2 h adopting a ramp rate of 2 or 30 °C/min, respectively.

Reaction products were characterized by X-ray diffraction (XRD) analysis using a Philips diffractometer and Cu Kα radiation. The mass fraction of anatase and rutile was determined using Equations (1) and (2) [24,25]:

Anatase (%) = [0.79I_A_/(0.79I_A_ + I_R_)] × 100 (1)

Rutile (%) = {1/[(0.79I_A_ + I_R_)/I_R_]} × 100, (2)

where I_A_ and I_R_ are the peak intensities of [101] and [110] reflections for anatase and rutile, respectively. Differential thermal analysis (DTA) and thermo-gravimetric analysis (TGA) were performed in air with a heating rate of 10 °C/min using a Netzsch model 409 thermoanalyzer (Selb, Germany) and α-alumina as reference material. The chemical structure of the prepared particles was examined by using a Fourier transform infrared spectrophotometer (FTIR, Bruker, IFS-88, Billerica, MA, USA) in the range from 4000 to 600 cm^−1^ with a 2 cm^−1^ resolution.

The specific surface area was measured by the BET method utilizing N_2_ as adsorbed after drying at 80 °C for 12 h. The average pore diameter was estimated by the desorption branch of the isotherm and the BJH formula, while the total pore volume was obtained from the volume of N_2_ adsorbed at P/P_0_ = 0.995. The morphologies of synthesized products were examined, after gold coating, by scanning electron microscopy (SEM), using a Philips microscopy (XL series, Almelo, The Netherlands) equipped by energy-dispersive X-ray spectroscopy (EDX) for the elemental analysis.

## 3. Results

### 3.1. Thermal Behaviour of the Xerogels

**Figure 1:**
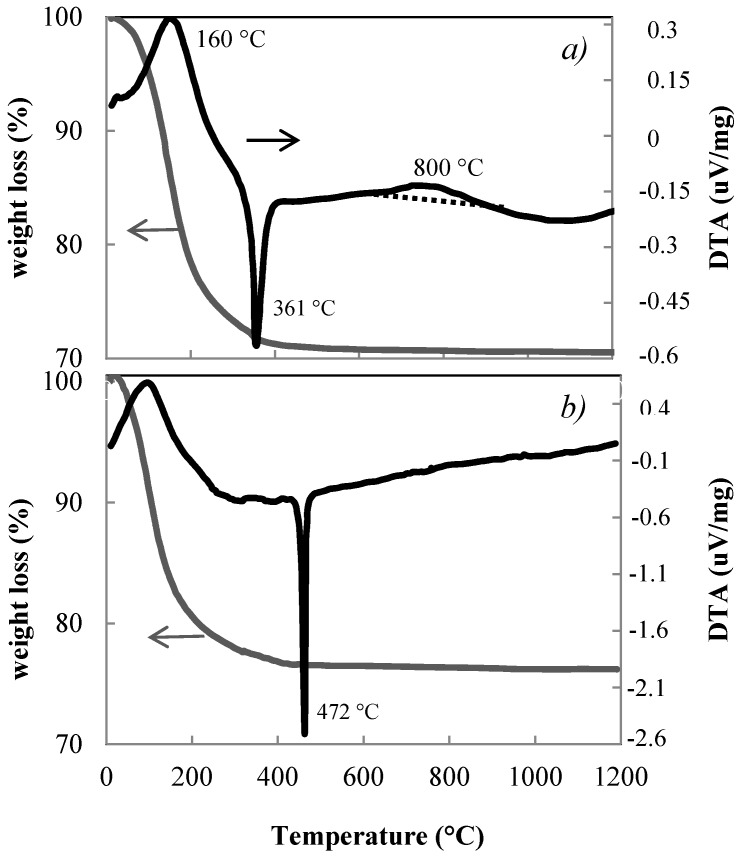
DTA and TGA of xerogels of pure titania (**a**) and of titania containing 5 mol % of zirconia (**b**).

The xerogels obtained by hydrolysis at room temperature of both Ti(OC_2_H_5_)_4_ and mixtures of Ti(OC_2_H_5_)_4_ with Zr(OC_3_H_7_)_4_ (5 mol %) both catalysed with different concentrations of NH_4_OH resulted in XRD amorphous xerogels. Both pure titania and titania containing 5 mol % of zirconia xerogels show a broad endothermic DTA peak with maximum around 160 °C due to water loss (Figure 1a,b). The exothermic peak at 361 °C for the pure titania xerogel is due to the crystallization of anatase (Figure 1a), while such transformation for the xerogel with 5 mol % of zirconia takes place at higher temperature with a strong and sharp peak at 472 °C (Figure 1b). Pure titania xerogel shows a very broad exothermic peak between 600 °C and 900 °C with a maximum at 800 °C attributable to the anatase–rutile transformation. This peak is absent for the xerogel containing 5 mol % of zirconia confirming that zirconia inhibits the transformation of anatase into rutile [26]. 

### 3.2. Peptization of TiO_2_ Xerogels with HNO_3_

#### 3.2.1. Effect of the Concentration

**Figure 2:**
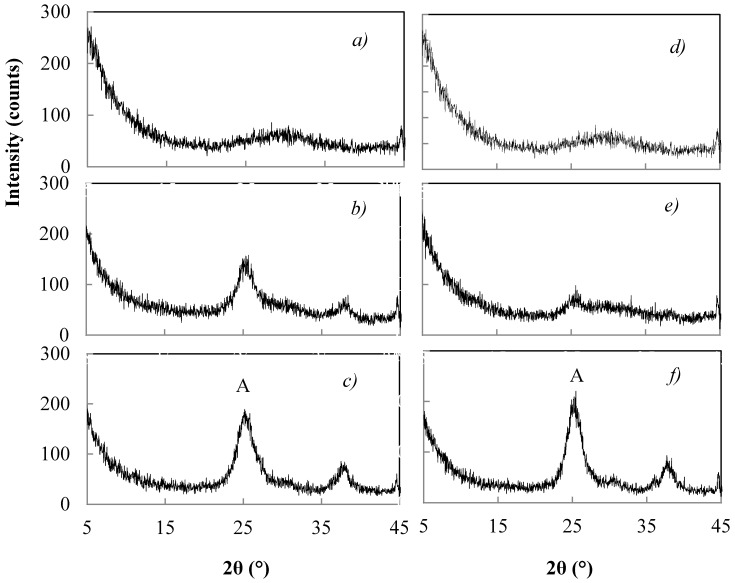
XRD patterns of products hydrolyzed for 1 h in the presence of NH_4_OH 0.07 M (**a**–**c**) or 1.0 M (**d**–**f**) and subsequently treated for 1 h with HNO_3_ 0.02 M (**b**,**e**) or 0.1 M (**c**,**f**). (A = anatase).

The peptizing treatment at 50 °C of TiO_2_ xerogel at pH = 1 in the presence of 0.1 M HNO_3_ favours the crystallization of anatase with traces of brookite, while at pH = 6.5 with 0.02 M HNO_3_ little crystallization of anatase results. The XRD patterns of Figure 2, show the xerogels catalysed for 1 h with 0.07 M NH_4_OH (Figure 2a–c) or 1.0 M NH_4_OH (Figure 2d–f), and comparisons with un-peptized (Figure 2a,d) and peptized samples for 1 h in the presence of 0.02 M HNO_3_ (Figure 2b,c) or 0.1 M HNO_3_ (Figure 2e,f). An amorphous product results without the peptizing treatment (Figure 2a,d), while increasing crystallinity of anatase results for samples peptized with increasing concentrations of HNO_3_ (Figure 2b,c,e,f). When the peptizing treatment time was either longer than 5 h or in the presence of HNO_3_ with a concentration higher than 0.1 M, the dissolution of the products resulted.

**Figure 3:**
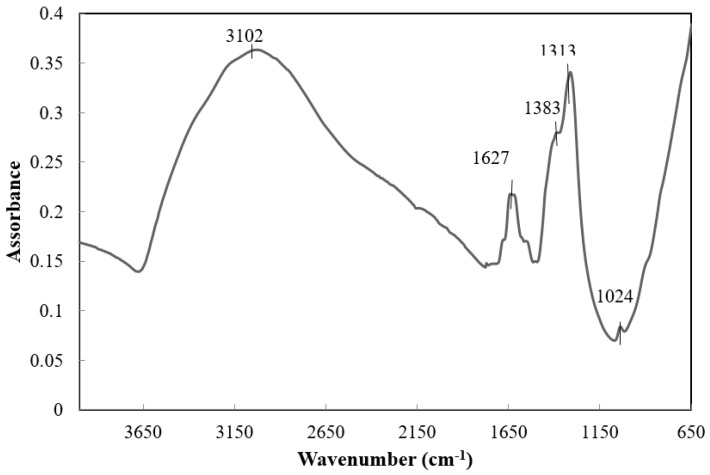
FT-IR spectra of product hydrolyzed for 1 h in the presence of NH_4_OH 1.0 M and subsequently treated for 1 h with 0.1 M HNO_3_.

The presence of nitrogen in the peptized samples has been detected by IR spectra (Figure 3) in which the asymmetric stretching band of NO_3_^−^ ion at 1383 cm^−1^ appears together the broad absorption peak at 3102 cm^−1^ related to a stretching vibration of the O–H group and a peak at 1627 cm^−1^ assigned to water.

#### 3.2.2. Effect of Treatment Time of Peptization 

The treatment times of peptization of titania gel under stirring were 1 or 3 h, respectively. The crystallinity of the resulting anatase with traces of brookite was practically unaffected by both the concentration of the hydrolyzing NH_4_OH and by the treatment time of the gel when a constant concentration of HNO_3_ was used. 

The crystal sizes of these samples were determined from the line broadening of [101] peak of anatase using the Sherrer formula. The resulting average crystal sizes and the corresponding polymorphic form of titania are reported in Table 1. Crystal sizes do not vary much, only about 4.7 nm, and a trace of brookite was found in all samples. 

In order to obtain the formation of mesoporous titania, a thermal treatment of both un-peptized and peptized products must be performed. It assures both the complete crystallization of titania and a certain amount of coarsening of the very small crystals of anatase resulting from the peptizing treatment.

### 3.3. Thermal Treatments of Peptized Titania

After calcination at 450 °C the peptized samples show IR spectra characterized by a significant decrease in both the OH group and water band intensities. The band of NO_3_^−^ ion at 1383 cm^−1^ also significantly decreases after calcination. 

Table 2 summarizes the characteristics of the products resulting from the hydrolysis of Ti(OC_2_H_5_)_4_ for 1 h in the presence either 0.07 or 0.1 M NH_4_OH that is subsequently peptized for 1 or 3 h with 0.02 M or 0.1 M HNO_3_, and finally heat treated for 2 h at different temperatures up to 600 °C using ramp rates of 2 °C or 30 °C·min^−1^, respectively.

#### 3.3.1. Sample Hydrolysed with 0.07 M NH_4_OH and Peptized with 0.02 M HNO_3_

**Figure 4:**
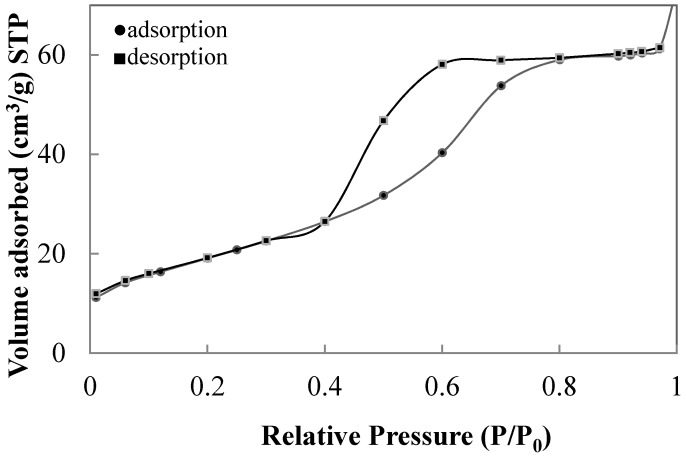
N_2_ absorption-desorption curves of the sample T1 reported in Table 2.

When the hydrolysis is catalysed in the presence of 0.07 M NH_4_OH and peptized with 0.02 M HNO_3_, respectively, only anatase crystallizes after calcination at 450 °C for 2 h (sample T1). This sample has a mesoporous structure according to the hysteresis of the N_2_ absorption-desorption curves shown in Figure 4. This sample is characterized by a surface area of 39.4 m^2^/g, crystal size of 21.9 nm and an average pore diameter of 6.6 nm measured using the desorption branch of N_2_ by the isotherm using BJH formula. 

#### 3.3.2. Samples Hydrolysed with 0.07 M NH_4_OH and Peptized with 0.1 M HNO_3_

**Figure 5:**
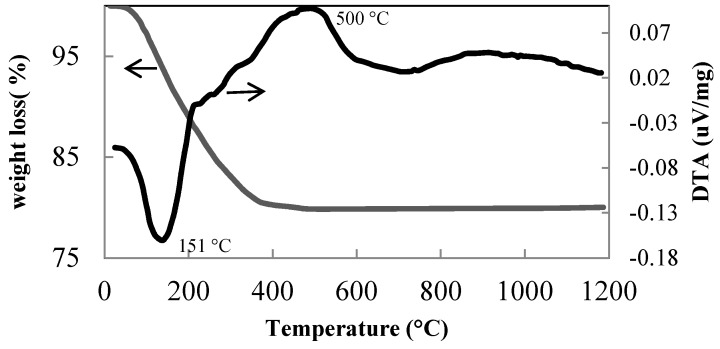
DTA-TGA curves of A sample reported in Table 1.

The precursor of these samples, corresponding to the A sample listed in Table 1, contains anatase with very small crystal size and traces of brookite. The DTA and TGA (Figure 5) of this sample shows a broad exothermal peak at very low temperatures, in the range between 300 °C and 600 °C with a maximum at 500 °C, which is due to the anatase–rutile transformation. The large endo-thermal peak with maximum at 151 °C can be attributed to the water loss prevalently related to both adsorbed water and hydroxyls decomposition. The weight loss at higher temperature can be related either to further de-hydroxylation and/or to nitrate evolution adsorbed during the peptizing treatment.

**Figure 6:**
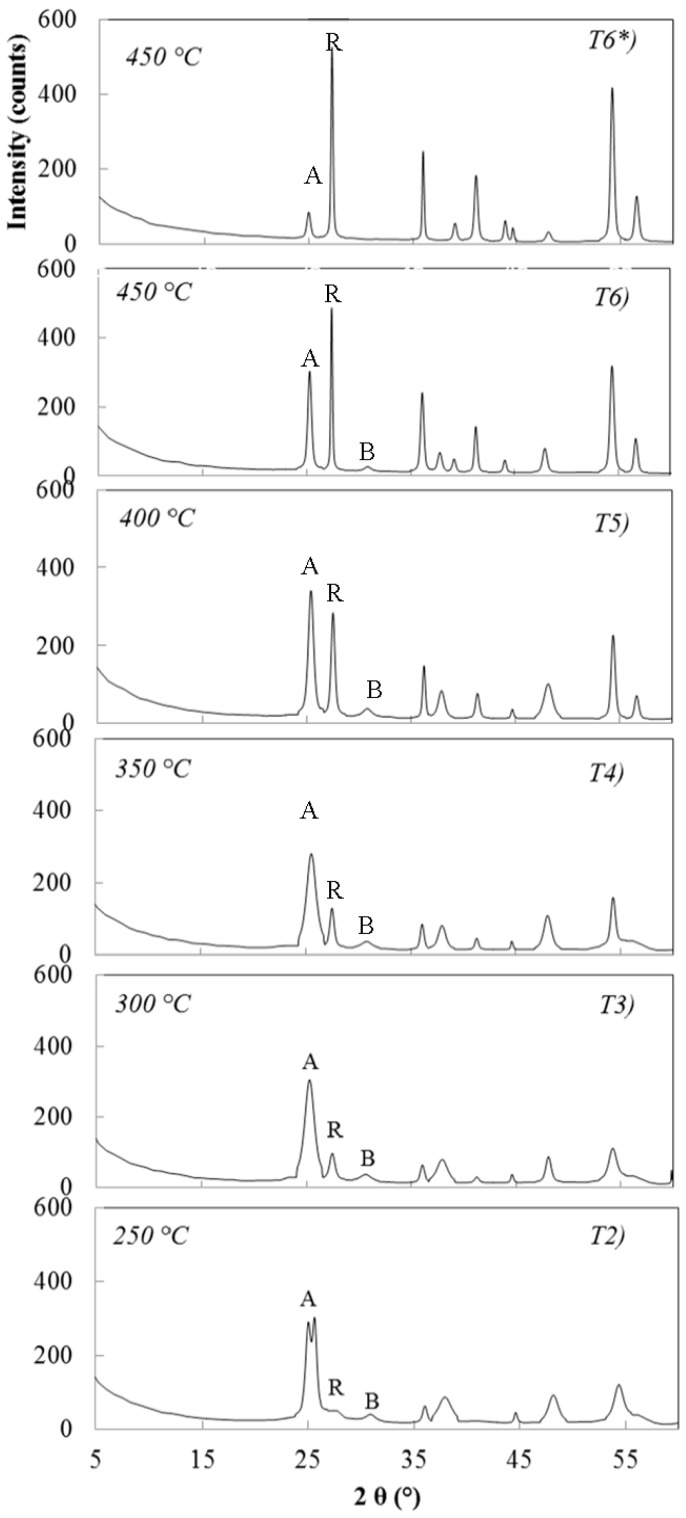
XRD patterns of samples T2, T3, T4, T5, T6, T6* reported in Table 2. (A = anatase; R = rutile; B = brookite).

The samples T2, T3, T4, T5 and T6 (Table 2) differ only in the calcination temperature of precursor A (Table 1). Calcination temperatures varied between 250 °C and 450 °C. The sample was firstly treated for 2 h at 250 °C and cooled at room temperature (Sample T2), then it was re-treated for 2 h at 300 °C and cooled (Sample T3). Analogous heat treatments were performed at 350 °C (Sample T4), 400 °C (Sample T5) and 450 °C (Sample T6), respectively. It can be seen from the XRD patterns of Figure 6 that at 250 °C crystalline anatase is the main phase with traces of rutile and brookite. At 300 °C, 350 °C and 400 °C the anatase–rutile transformation takes place while at 450 °C rutile is the main crystalline phase. Traces of brookite persist up to 450 °C. Brookite disappeared when the same precursor was directly treated at 450 °C for 2 h using a ramp rate of 2 °C/min (Sample T6*). On the other hand, such thermal treatment reduces the anatase content from 30.1 (Sample T6) to 9.0% (Sample T6*). 

**Figure 7:**
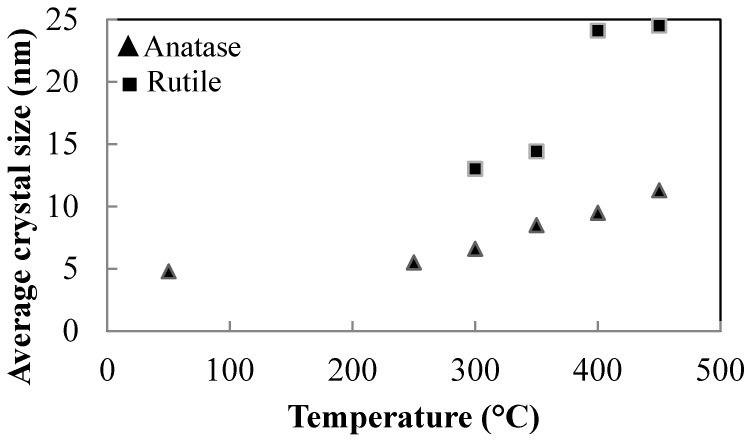
Average crystallite size of sample A listed in Table 1 at increasing temperature of calcination.

Increasing calcination temperature, the anatase crystal sizes change from 4.8 nm for the un-calcined precursor to 11.3 nm for the 450 °C calcined sample. Rutile shows significant grain growth as the anatase to rutile transition proceeds. The crystal size changes from 13.0 nm for the sample thermally treated at 300 °C, to 24.5 nm after heat treatment at 450 °C (Figure 7). The detected anatase–rutile transformation indicates that the peptizing treatment with 0.1 M HNO_3_ is sufficient to promote this transformation at very low temperatures [24].

We must take into account that after the peptizing treatment with HNO_3_, the products were not washed with deionized water but they were directly dried in an oven at 60 °C. 

#### 3.3.3. Samples Hydrolysed with 1.0 M NH_4_OH and Peptized with 0.02 M HNO_3_

The samples T7, T8, T9 and T10 of Table 2 form only anatase after the treatments of calcination at 450 °C.

In this case the concentration of the peptizing HNO_3_ appears to be insufficient to promote the anatase–rutile transformation. 

It must be pointed out that anatase crystals bigger in size result with a ramp rate of 30 °C/min^−1^ (T8 and T10 samples) compared with smaller crystals when a ramp rate of 2 °C/min^−1^ was adopted (T7 and T9 samples).

The effect of the treatment time with the peptizing HNO_3_ on the anatase crystal size is not clear. An increase of the crystal sizes of anatase results with longer peptizing treatment and a calcination ramp rate of 2 °C/min (T7 and T9 samples) while a certain reduction can be seen with a ramp rate of 30 °C/min (T8 and T10 samples) as reported in Table 2. 

**Figure 8:**
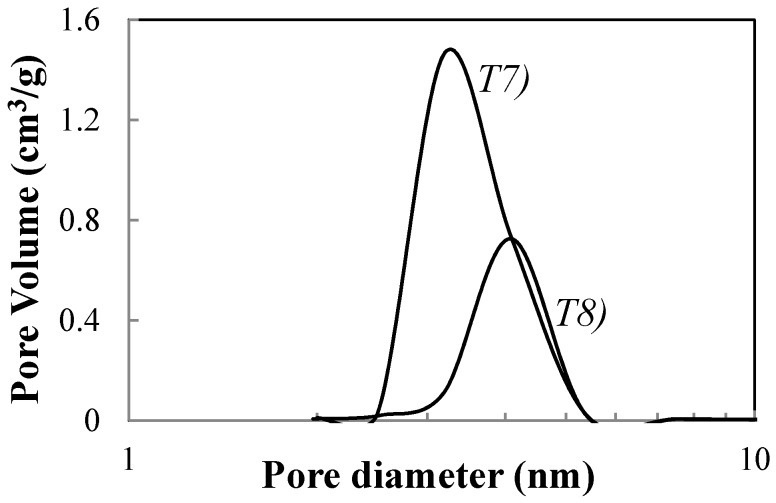
Pore size distribution of samples T7 and T8 determined according to BJH formula in desorption brunch of N_2_.

The increase of the crystal sizes of anatase samples involves a decreases in surface area and a corresponding increase of the average sizes of mesopores. This last feature is emphasized comparing the pore sizes distribution of T7 and T8 samples (Figure 8) determined from the BJH formula in the N_2_ desorption branch.

#### 3.3.4. Sample Hydrolyzed with 1.0 M NH_4_OH and Peptized with 0.1 M HNO_3_

**Figure 9:**
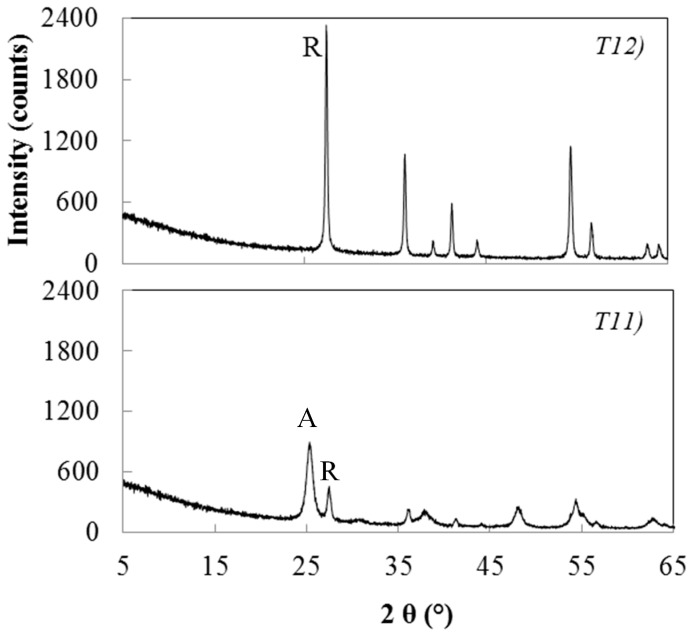
XRD patterns of sample T11 and T12. (A = anatase; R = rutile).

The samples T11, T12, T13, T14 and T15 listed in Table 2 have been obtained in the presence of the highest concentrations of both catalysing and peptizing agents. Also in this case, the calcination of these samples peptized with 0.1 M HNO_3_ favours the crystallization of rutile. The comparison between the XRD patterns of the sample T11, heat treated for 2 h at 450 °C, with that of the sample T12, heat treated for 2 h at 600 °C (Figure 9), shows an increase of rutile content from 65% to 100%.

The higher ramp rate adopted up to the temperature of calcination also favours the formation of rutile as results by comparing T11 and T14 samples with T13 and T15 samples (Table 2). On the contrary, the crystallization of anatase is favoured when the treatment time with the peptizing HNO_3_ is increased (T13 and T14 samples in Table 2). In all of the calcined samples containing mixtures of anatase and rutile, the crystallite sizes of the rutile phase are on average twice that of anatase, confirming that the transition of anatase to rutile is generally accompanied by significant grain growth [27,28]. The calcined products listed in Table 2 are characterized by average pore diameter on order of 3–5 nm.

### 3.4. Hydrolysis, Peptization and Calcination of Titania in Mixture with Zirconia (5 mol %)

In Table 3 the crystal sizes and polymorph phases of titania are summarized for the products obtained by co-hydrolysis of Ti(OC_2_H_5_)_4_ in mixture with Zr(OC_3_H_7_)_4_ (5 mol %).

These products have been catalysed for 1 h in the presence of 1.0 M NH_4_OH and peptized or not with different concentration of HNO_3_ for 1 h or 3 h followed by heat treatment for 2 h at 450 °C or 600 °C with a ramp rate of 2 °C or 30 °C min^−1^, respectively.

**Figure 10:**
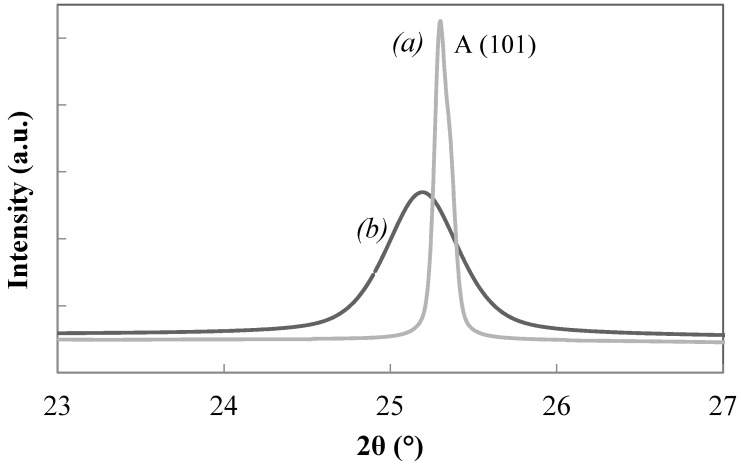
Anatase peak (101) of pure titania (*a*), T9 sample in Table 2, and of titania–zirconia (5 mol %) solid solution (*b*), sample H in Table 3, both calcined at 450 °C.

**Figure 11:**
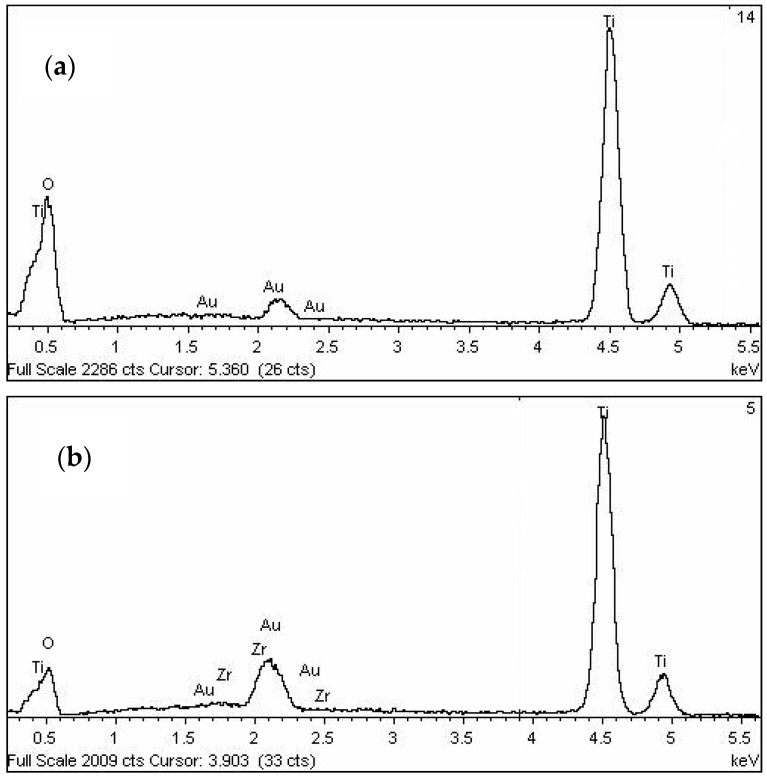
EDX analysis of T9 (**a**), and H (**b**), samples.

The presence of Zr in titania–zirconia (5 mol %) solid solution is confirmed by the shift at low angle of the maximum of (101) peak of anatase ((*b*) in Figure 10) compared to that of pure titania ((*a*) in Figure 10). This behaviour is due to bigger ionic radius of Zr (0.086 nm) respect to that of Ti (0.068 nm). In addition it is well known that Zr promotes a reduction of titania crystallite size [29,30] as it can be seen from larger line broadening of solid solution main peak (Figure 10). The presence of Zr in solid solution is also confirmed comparing in EDS analysis (Figure 11) the same samples reported in Figure 10. 

It can be seen that after calcination only anatase is present with crystal sizes that are affected by several features (Table 3):

1. The calcination of the unpeptized samples favours the crystallization of anatase with relatively larger crystals (samples E and F).
2. Upon calcination, the peptized samples form anatase with crystals that are relatively smaller in size (samples G, H, I, J, L, M).
3. The peptizing treatment with increasing concentration of HNO_3_ reduces the anatase crystal size for samples G, I and L, which were subjected to the same peptizing time of 1 h and to the same heat treatment for 2 h at 450 °C.
4. The heat rate in calcination significantly affects the crystal size of anatase. Comparing sample L, heated at 2 °C/min, with sample N heat treated at 30 °C/min, very different crystal sizes have been detected. For sample L, the average crystal size was 6.3 nm, while for sample N it was 17.0 nm.
5. After calcination at either 450 °C or 600 °C, no anatase-to-rutile phase transformation has been detected. This confirms the inhibitory effect of zirconia on the anatase-to-rutile phase transition observed in the literature [26].
6. Larger anatase crystal sizes result for products thermally treated at 600 °C, compared with those treated at 450 °C (Samples E, F and L, M).

### 3.5. Morphology of Un-Peptized, Peptized and Calcined Products

**Figure 12:**
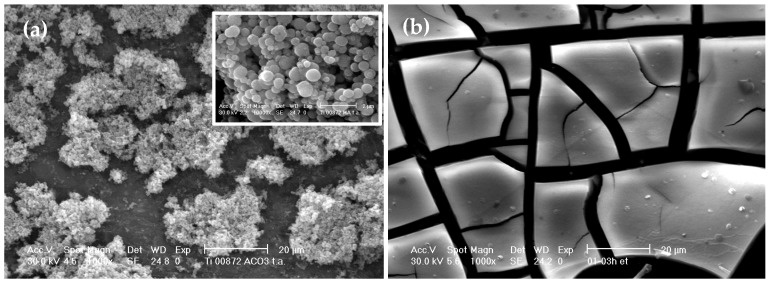
SEM micrographs of un-peptized sample (**a**) and of the same product peptized for 3 h with 0.1 M HNO_3_ (**b**).

SEM micrographs reported in Figure 12 show the morphology of both un-peptised sample (Figure 12a) obtained by hydrolysis catalysed for 1 h in the presence of 1.0 M NH_4_OH and of the corresponding product peptized for 3 h with 0.1 M HNO_3_ (Figure 12b), both dried in air at 60 °C. The unpeptised sample show spherical and poor agglomerated particles of amorphous titania (Figure 12a). Owing the 3 h peptization treatment the starting spherical particles disappear and a very homogenous texture results as a consequence of the complete agglomeration among the particles (Figure 12b). The homogeneous texture corresponds to the crystallized anatase according to XRD analysis. No significant differences in the morphology between peptized and the corresponding calcined samples have been detected. All the products obtained after the calcination are characterized by a heterogeneous particle size distribution with average particle sizes of a few microns. Rarely, particles below 1 µm in size have been observed. 

### 3.6. Effect of Drying Method on the Aggregation of Titania Particles

**Figure 13:**
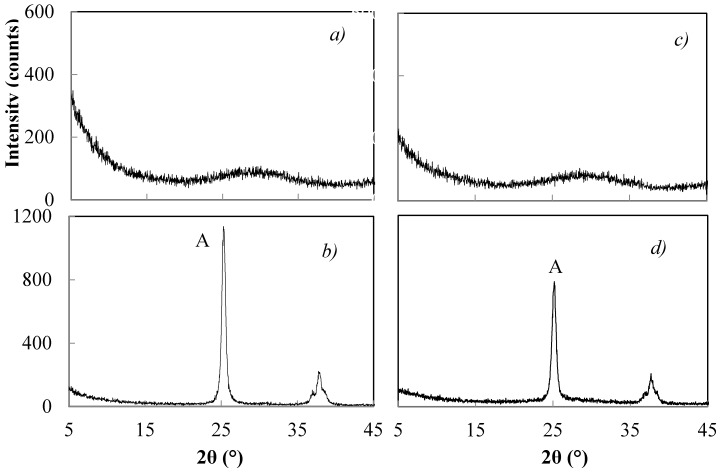
XRD patterns of sample hydrolysed at r.t. for 1 h with 1.0 M NH_4_OH, peptized for 1 h with 0.02 M HNO_3_, dried by lyophilization (**a**,**b**) or at 60 °C (**c**,**d**) and calcined for 2 h at 450 °C with a ramp rate of 30 °C/min (**b**,**d**). (A = anatase).

**Figure 14:**
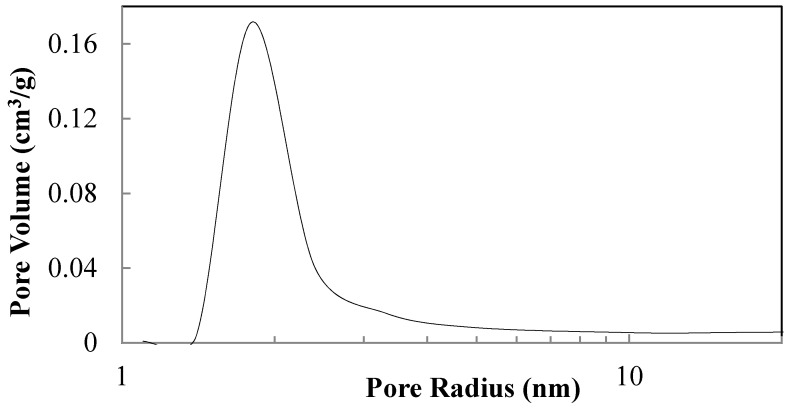
Pore size distribution of sample hydrolysed at r.t. for 1 h with 1.0 M NH_4_OH, peptized for 1 h with 0.02 M HNO_3_, dried by lyophilization and calcined for 2 h at 450 °C with a ramp rate of 30 °C/min.

**Figure 15:**
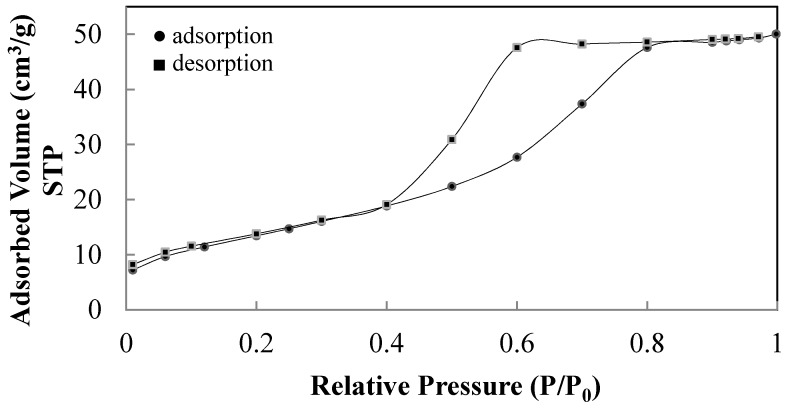
N_2_ adsorption–desorption curves of sample hydrolysed at r.t. for 1 h with 1.0 M NH_4_OH, peptized for 1 h with 0.02 M HNO_3_, dried at 60 °C and calcined for 2 h at 450 °C with a ramp rate of 30 °C/min.

The drying method of the peptized precursors affects the aggregation of titania particles, which in turn determines the type of porosity before and after calcination. Peptization with a dilute concentration of HNO_3_ results in amorphous materials (Figure 13a,c) with a BET surface area of 328.3 m^2^/g for the lyophilized and 287.0 m^2^/g for the 60 °C air-dried sample, while, after calcination, crystalline anatase is obtained with crystal sizes of 24.6 nm for the lyophilized sample compared to 19.8 nm for the heat-dried sample (Figure 13b,d). The calcination of the lyophilized sample produces a microporous structure (Figure 14), while the heat-dried and calcined sample is structurally micro-/mesoporous (Figure 15). Correspondingly, BET surface areas decrease to 52.2 and 19.3 m^2^/g, respectively. 

**Figure 16:**
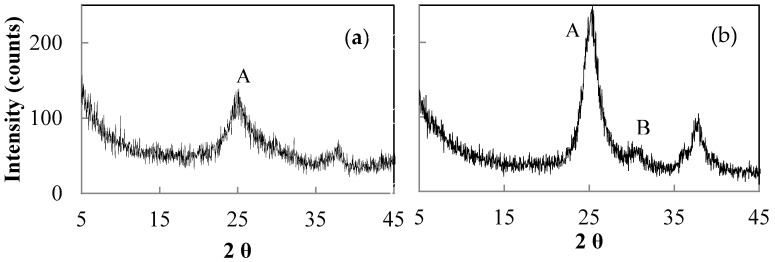
XRD patterns of sample hydrolysed at r.t. for 1 h with 1.0 M NH_4_OH, peptized for 1h with 0.1 M HNO_3_, dried by lyophilization (**a**) or at 60 °C (**b**) and calcined for 2 h at 450 °C with a ramp rate of 30 °C/min (**b**,**d**). (A = anatase; B = brookite).

As previously mentioned, anatase–rutile mixtures result when the calcination is performed on precursors peptized with 1.0 M HNO_3_. The peptized precursors, dried by lyophilization or at 60 °C, contain nanometer anatase with a crystallite size of 4.5 and 5.2 nm, respectively (Figure 16a,b). After calcination, the lyophilized sample is characterized by bigger crystallite sizes and, consequently, by a smaller anatase–rutile molar ratio compared to the heat-dried sample.

**Figure 17:**
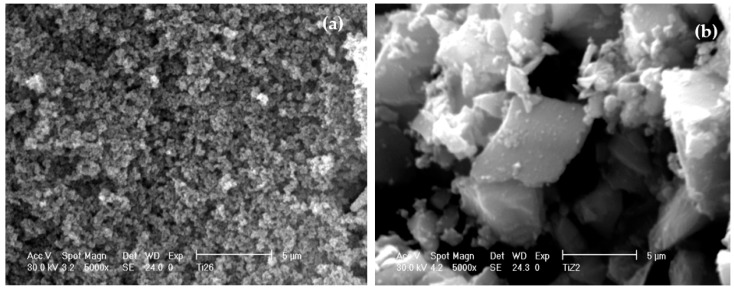
SEM micrographs of the sample hydrolysed at r.t. for 1 h with 0.07 M NH_4_OH, peptized for 1h with 0.05 M HNO_3_, dried by lyophilization (**a**) or at 60 °C (**b**) and calcined for 2 h at 450 °C.

Morphological differences between lyophilized and heat treated samples have been observed after calcination. The lyophilized sample (Figure 17a) shows a minor aggregation among titania particles compared with the same precursor dried by heat treatment (Figure 17b). Consequently, interparticle macropores of titania can be detected for the lyophilized sample while the sample dried by heat treatment manifests micrometric titania aggregates characterized by intraparticle mesopores.

## 4. Discussion

It is well known that rutile polymorph is the stable phase of titania at room temperature, while anatase and brookite are thermodynamically metastable [21]. The existence of anatase at room temperature is due to the fact that it is kinetically stabilized at room temperature [26]. The transformation upon calcination of amorphous titania to crystalline anatase takes place in the temperature range between 350 °C and 450 °C, while the anatase–rutile transformation occurs between 600 °C and 1100 °C [31]. This last transformation is affected by oxygen defect levels [32], which in turn depend on the impurities, inhibitors or promoters of the anatase-to-rutile phase transition. Nitrogen as a TiO_2_ dopant is a typical promoter of the anatase-to-rutile phase transformation [33,34]. The peptizing treatment of un-doped titania xerogels of with 0.1 M HNO_3_ at pH = 1 favours the crystallization of anatase with crystallites very small in size on the order of 4.6 nm, while amorphous products results when the same treatment is performed with diluted solution of HNO_3_, corresponding to pH = 6.5. The calcination of pure titania xerogel or of the corresponding peptized samples with diluted solution of HNO_3_ promotes the crystallization of anatase with crystals larger in size than those corresponding xerogel peptized in the presence of 0.1 M HNO_3_. This result can be justified in terms of different reactivity. The higher reactivity on calcination of amorphous precursors justifies the formation of anatase with crystals larger in size compared to those of crystalline precursors resulting after peptization with 0.1 M HNO_3_. It is well known that a higher initial primary particle size reduces the grain growth rate of crystals with increasing temperature [31]. The precursors peptized in the presence of diluted HNO_3_ do not show the anatase–rutile transformation after calcination either when the precursors have been peptized for different times (1 or 3 h) or when ramp rates of 2 °C or 30 °C/min were adopted. In these circumstances shorter times of peptization with diluted HNO_3_ favour the formation of anatase crystals larger in size compared with the smaller ones detected for longer treatment times. In the last case the dissolution of more reactive particles of the xerogel is favoured, with consequential reduction of the crystal growth of anatase. The high calcination ramp rate also enhances the formation of large anatase crystals due to a possible nucleation and crystal growth mechanism during the anatase crystallization. A high ramp rate involves a shorter time in the temperature range of nucleation, thus reducing the concentration of the nuclei with consequent formation of crystals of larger size. This behaviour contrasts with the results obtained on calcination of amorphous precursors showing a reduction of the titania crystal size when a high heating rate was adopted [35].

The calcination of the precursors obtained from titania xerogel peptized in the presence of 0.1 M HNO_3_ gives rise to the anatase-to-rutile transformation. According to [36,37], the temperature of the anatase-to-rutile phase transformation decreases with a smaller crystal size of the anatase precursor. Such behaviour is confirmed by the lowest temperature transition (250 °C) detected for precursors with crystal size of anatase of order of 4.6 nm obtained by peptization of titania xerogel in the presence of 0.1 M HNO_3_. The tendency to form rutile is also enhanced by high temperature of calcination, high ramp rate up to the calcination temperature and finally low treatment time of peptization. Such parameters allow us to obtain mixtures of anatase-rutile in different amounts and with variable crystallite sizes. The peptizing treatments of titania xerogels containing 5 mol % zirconia in the presence of increasing concentration of HNO_3_ determine, in a similar way to pure titania xerogel, precursors characterized on calcination by a high crystallinity but with anatase crystals decreasing in size. Both unpeptized and peptized samples, both thermally treated at 600 °C, do not form rutile confirming the inhibitor role of zirconia in the anatase–rutile transformation [26]. In this circumstance, the inhibitor effect of zirconia prevails over the promoter effect of nitrogen doping.

The aggregation of titania particles that takes place either under stirring during the hydrolysis or the drying method of the corresponding precursors affects both the crystal and pore size of crystallized phases upon calcination. The formation of the mesoporous structure of titania appears to be significantly related to the drying method; in particular no significant mesopores result when drying is performed by lyophilization or in air at room temperature. On the contrary, the drying by heat treatment at 60 °C appears to favour a mesoporous structure. The mesoporous products are in the micrometers in size and show an easy tendency toward sedimentation when dispersed in aqueous suspensions [7]. Such a feature is very useful in catalysis production because an easy, recyclable process can be performed. A few minutes are sufficient for the quiescent sedimentation of the calcined products with a slight opalescent supernatant persisting for a few hours. In comparison, the commercial product P25 (Degussa) requires longer sedimentation with a milky opalescence that persists for several hours.

## 5. Conclusions

Micrometer aggregates of mesoporous titania based on anatase or anatase–rutile mixtures have been prepared from TiO_2_ precursors peptized with 0.1 M HNO_3_ (pH = 1) at 50 °C and subsequently calcined. Nitrogen in HNO_3_ has proved to be an effective promoter of anatase–rutile transformation. However, adequate HNO_3_ concentrations and peptizing times are required. In the presence of 0.1 M HNO_3_ the peptization time should not be less than 1/2 h and not more than 3 h. In the first case, the promoter effect of the anatase–rutile transformation is reduced, while in the latter the dissolution of the gel takes place especially when more concentrated HNO_3_ solutions are used.

The peptization promotes the formation of crystalline anatase with a very small crystal size on the order of 4.6–4.8 nm. The calcination of such peptized precursors determines the anatase–rutile transformation with a percentage ratio that can be changed with the crystal size of crystallized titania. The rutile formation is favoured by larger crystals, obtained by changing various parameters of synthesis, to control the crystal size of titania and the amount of crystalline rutile. When the precursor is peptized with diluted HNO_3_ solution, the rutile crystallizes only at high temperature, with a drastic reduction in both the surface area and catalytic activity. The rutile content from precursors peptized with 0.1 M HNO_3_ increases according to the following parameters: high concentration of catalysing NH_4_OH, low peptizing time, high temperature of calcination and high ramp rate up to calcination temperature. 

After calcination the TiO_2_ in mixture with 5 mol % ZrO_2_ up to 600 °C, only anatase is produced either for un-peptized or peptized precursors showing a prevalent inhibitor effect of zirconia on the anatase to rutile transition compared to the promoter effect of HNO_3_. Smaller anatase crystal sizes result for peptized precursors compared with un-peptized ones. High values of both the calcination temperature and the ramp rate favour large anatase crystal sizes.

The drying method of precursors before calcinations significantly affects the porous structure of both dried and calcined products. The agglomeration of titania particles takes place not only during the hydrolyzing step and peptizing treatment but it is also altered by the drying method. In particular, drying in air at room temperature and lyophilization appear unfavourable for the formation of mesoporous structure. Heat drying treatments form mesoporous structures. Before and after the calcination treatment all the products are characterized by a heterogeneous distribution of particles sizes of order of several micrometers. These particles are easily separated by sedimentation. Such a feature is very useful in catalysis preparation because an easy, recyclable process can be performed.

From these results, we can conclude that the objectives to obtain recyclable aggregates of mesoporous titania as anatase form or as anatase–rutile mixtures with different ratio and characterized by variable values of both crystallinity and surface area have been attained. The next objective is to select from these products those with appropriate characteristics to be tested as recyclable photocatalysts.

## Figures and Tables

**Table 1:** Crystal sizes and polymorphic forms of titania for the samples reported in Figure 3.

Sample	NH_4_OH Concentration/Treatment Time (M/h)	HNO_3_ Concentration/Treatment Time (M/h)	Crystal Size (nm) by XRD	Polymorphic Phases A (Anatase), B (Brookite)
A	0.07/1	0.1/1	4.8	A, B (traces)
B	0.07/1	0.1/3	4.7	A, B (traces)
C	1.0/1	0.1/1	4.7	A, B (traces)
D	1.0/1	0.1/3	4.6	A, B (traces)

**Table 2:** BET surface area, average pore diameter, crystal size and percentage of polymorphic phases of titania for products hydrolyzed for 1 h with NH_4_OH 0.07 M or 0.1 M, subsequently treated or not for 1 h or 3 h with HNO_3_ 0.02 M or 0.1 M, and finally heat treated for 2 h at different temperatures up to 600 °C using heating rates of 2 or 30 °C·min^−1^, respectively.

Sample	NH_4_OH Concentration/Treatment Time (M/h)	HNO_3_ Concentration/Treatment Time (M/h)	Calcination Temperature/ Heating Rate (°C/°C min^−1^)	BET Surface Area (m^2^/g)	Average Pore Diameter (nm)	Crystal Size by XRD (nm)	Polymorphic Phases (%) A (anatase) R (rutile)
T1	0.07/1	0.02/1	450/2	39.4	6.6	21.9	100 % (A)
T2	0.07/1	0.1/1	250/2	-	-	5.6	100 % (A)
T3	0.07/1	0.1/1	300/2	-	-	6.6 13.0	74.8 (A) 25.2 (R)
T4	0.07/1	0.1/1	350/2	-	-	8.5 14.4	67.5 (A) 32.5 (R)
T5	0.07/1	0.1/1	400/2	-	-	9.5 24.1	64.8 (A) 35.2 (R)
T6	0.07/1	0.1/1	450/2	-	-	11.3 24.5	30.1 (A) 69.9 (R)
T6*	0.07/1	0.1/1	450/2	-	-	15.1 26.4	A(9.0) R(91.0)
T7	1.0/1	0.02/1	450/2	70.5	3.4	13.8	100 (A)
T8	1.0/1	0.02/1	450/30	65.5	4.0	19.8	100 (A)
T9	1.0/1	0.02/3	450/2	62.7	5.2	20.4	100 (A)
T10	1.0/1	0.02/3	450/30	-	-	26.3	100 (A)
T11	1.0/1	0.1/1	450/2	46.8	3.3	12.2 24.1	64.9 (A) 35.1 (R)
T12	1.0/1	0.1/1	600/2	10.5	5.2	48.1	100 (R)
T13	1.0/1	0.1/1	450/30	70.4	5.1	20.4 37.2	22.8 (A) 77.2 (R)
T14	1.0/1	0.1/3	450/2	30.3	3.3	17.0 43.1	73.3 (A) 26.7 (R)
T15	1.0/1	0.1/3	450/30	-	-	25.5 45.5	43.7 (A) 56.3 (R)

**Table 3:** Anatase crystal sizes of samples obtained by hydrolysis of Ti(OC_2_H_5_)_4_ in mixture with Zr(OC_3_H_7_)_4_ (5 mol%) catalysed for 1 h in the presence of 1.0 M NH_4_OH and peptized or not with different concentration of HNO_3_ for 1 h or 3 h followed by heat treatment for 2 h at 450 °C or 600 °C with a ramp rate of 2 °C or 30 °C min^−1^, respectively.

Sample	NH_4_OH Concentration (M)/Treatment Time (h)	HNO_3_ Concentration (M)/Treatment Time (h)	Calcination Temperature (°C)/Heating Rate (°C min^−1^)	Crystal Size by XRD (nm)	Polymorphic Phase A (Anatase)
E	1.0/1	0/0	450/2	15.7	A
F	1.0/1	0/0	600/2	18.4	A
G	1.0/1	0.02/1	450/2	11.5	A
H	1.0/1	0.02/3	450/2	7.5	A
I	1.0/1	0.05/1	450/2	7.3	A
J	1.0/1	0.05/3	450/2	6.0	A
L	1.0/1	0.1/1	450/2	6.3	A
M	1.0/1	0.1/1	600/2	8.5	A
N	1.0/1	0.1/1	450/30	16.0	A
